# Intuitive thinking predicts false memory formation due to a decrease in inhibitory efficiency

**DOI:** 10.3389/fpsyg.2023.1195668

**Published:** 2023-09-22

**Authors:** Giorgio Gronchi, Stefania Righi, Gioele Gavazzi, Fiorenza Giganti, Maria Pia Viggiano

**Affiliations:** Psychology Section, Department of Neuroscience, Psychology, Drug Research and Child's Health, University of Florence, Florence, Italy

**Keywords:** DRM, false memories, CRT, dual process theory of thought, inhibition, cognitive reflection, reasoning

## Abstract

False memory formation is usually studied using the Deese-Roediger-McDermott paradigm (DRM), in which individuals incorrectly remember words that were not originally presented. In this paper, we systematically investigated how two modes of thinking (analytical vs. intuitive) can influence the tendency to create false memories. The increased propensity of intuitive thinkers to generate more false memories can be explained by one or both of the following hypotheses: a decrease in the inhibition of the lure words that come to mind, or an increased reliance on the familiarity heuristic to determine if the word has been previously studied. In two studies, we conducted tests of both recognition and recall using the DRM paradigm. Our observations indicate that a decrease in inhibitory efficiency plays a larger role in false memory formation compared to the use of the familiarity heuristic.

## 1. Introduction

Memory is an active reconstructive process, and as a result, it is susceptible to distortions and false memories. The study of false memories has been extensively explored in experimental psychology since the adoption of the Deese-Roediger-McDermott paradigm (DRM), which has become a standard method for investigating this phenomenon. In the DRM task, initially developed by Deese (1959) and later modified by Roediger and McDermott (1995), participants are presented with a list of words that are semantically related (e.g., mug, handle, coffee, etc.), converging on a non-presented word that is semantically associated (referred to as a critical lure, such as the word ‘cup’ in our example). Following a delay, participants are asked to recall or recognize these words. In the recognition memory version, participants are required to determine if they remember words presented earlier, including the semantically related critical lure words (i.e., cup) that were never actually presented, among new words that are semantically unrelated to the studied words (e.g., doctor). Typically, participants mistakenly recognize or recall the critical lures as words that were included in the studied list (Roediger and McDermott, 1995). This false memory effect has been extensively documented in a substantial body of literature (see Gallo, 2010, for a review), including its cultural variations (Wang et al., 2021), and is commonly explained by the activation-monitoring theory (Roediger et al., 2001).

This theory is based on the notion of spreading activation in semantic memory networks (Collins and Loftus, 1975). Semantic concepts are represented as nodes interconnected through bidirectional associative links (whose strength is stronger when nodes belong to the same semantic category). Activation of a node spreads along the links to associated nodes within the network. In the DRM task, during encoding, both the presented words and critical lures (i.e., unstudied but semantically related words to the studied ones) are strongly activated through spreading activation from the presented words to the critical lures. During the recall and/or recognition phase, participants engage in a controlled source monitoring process to differentiate between studied and non-studied words. Incorrect retrieval or recognition of unstudied lures can be reduced through a conscious, controlled process of rejecting recollection (Brainerd et al., 2003). Hence, false memories might occur because subjects have insufficient source monitoring that fails to inhibit the indirect spreading activation of the critical lure. This implies that not all subjects are equally predisposed to memory illusion, and the investigation of how individual differences may modulate false memory production is still an open field of research (Winograd et al., 1998; Baird, 2001; Watson et al., 2005). A higher proneness to memory distortion has been observed in subjects with higher tendencies to dissociative experiences (Winograd et al., 1998), higher vividness of imagery (e.g., producing a photograph-like mental picture, Winograd et al., 1998), higher expertise in the domain of the material to be learned (Baird, 2001) and poor sleep (Malloggi et al., 2022a,b, 2023).

However, individual differences based on thought and reasoning have been little studied: for example, it has been observed that false memories are associated with convergent thinking (Dewhurst et al., 2011), categorization ability (Hunt and Chittka, 2014) and negatively correlated with fluid and crystallized measures of intelligence (Zhu et al., 2010). A more prolific line of research has investigated the relationship between Need for Cognition (NFC) (Graham, 2007; Leding, 2011, 2013; Wootan and Leding, 2015; Parker and Dagnall, 2018) and false memories. NFC (Cacioppo and Petty, 1982) can be defined as the tendency to look for challenging cognitive activities and enjoy such mentally effortful tasks. Results support the view that high-NFC individuals are prone to producing more true and false memories than those with low-NFC because of their tendency to greater elaboration of the stimuli. In the context of activation-monitoring theory, greater elaboration leads to stronger connections among nodes in the semantic memory network (Viggiano et al., 2006; Graham, 2007; Zaragoza et al., 2011).

There is also some correlational evidence about the association between false memories and intuitive thinking, which is the tendency to reach decisions quickly based on automatic processes within the dual process theory of thought. Gronchi et al. (2016) found that individuals who rely on intuition are more likely to produce false memories in a recognition memory prose task. This result has been recently replied to by Nichols and Loftus (2019) who employed a recognition DRM-task. However, these studies have critical limitations. First, only recognition tasks were employed. In order to disambiguate different explanations about the relation between thinking and false memories, it is also useful to employ a recall task, as described in Section 3 (see also Gronchi et al., 2016). Second, only correlational evidence has been provided in the studies of Gronchi et al. (2016) and Nichols and Loftus (2019); an experimental design is needed to exclude the possibility that the observed association may be due to confounding variables. Based on the above, we investigated whether and to what extent intuitive thinking influences the production of false memories by using an experimental design employing both recall and recognition tasks.

## 2. Analytical versus intuitive thinking

Empirical evidence has supported the long-standing philosophical distinction of two different systems of thought, known as the dual process theory of thought, for the last 30 years. The first system is fast, automatic, relatively effortless, and based on associative processes. The second system is slow, controlled, requires a certain amount of effort, and is based on deliberation (Sloman, 1996, 2014; Epstein and Pacini, 1999; Lieberman, 2003; Stanovich, 2004; Kahneman and Frederick, 2005; Evans, 2006; Pennycook et al., 2018). The dual process theory of thought is a general label that includes several specific theories that differ in some respects as to the interaction between the two systems (De Neys and Glumicic, 2008; Thompson, 2013; Sloman, 2014). Depending on the specifics that different authors emphasized, the two systems have been labeled in different ways: System 1 vs. System 2 (Stanovich, 1999; Stanovich, 2004; Kahneman and Frederick, 2005); intuition vs. deliberation (Sloman, 2014); intuitive vs. analytical (Epstein et al., 1996); associative vs. rule-based thinking (Sloman, 1996); and fast vs. slow thinking (Kahneman, 2011). Following Evans (2006), there are two main views about how the two systems may interact. The Default-Interventionist (DI) models sustain that System 1 generates responses by default and System 2 may or may not serially intervene (Evans and Stanovich, 2013). On the contrary, Parallel models claim that the two processes occur simultaneously with continuous monitoring of potential conflicts (Denes-Raj and Epstein, 1994; Sloman, 1996). In both cases, System 2 may suppress the response activated by System 1. A third, more recent, view sustains a Hybrid Two-Stage model (De Neys and Glumicic, 2008; Thompson, 2013; Newell et al., 2015). According to this model, a “shallow analytic monitoring process” is continuously active to detect potential conflicts between the responses produced by the two systems. When an actual conflict is found, an “optional deeper processing stage” is activated to inhibit the System 1 (intuitive) response.

Overall, dual process theories of thought reflect the general two-system views (stimulus-driven vs. goal-driven) that are recurrent across different psychology domains. In particular, the inhibitory mechanism hypothesized by dual process-theories of thought appears to be similar to inhibitory control within attention in perceptual tasks (Giovannelli et al., 2016; Gavazzi et al., 2017, 2019; Benedetti et al., 2020). Although the pioneering research of Kahneman, which paved the way to the dual-process distinction has its roots in the attention domain (Kahneman, 1973, 2011), the relationship between inhibition in thinking and inhibition in cognitive control is still an open problem (Dorigoni et al., 2022).

Another line of research (Shenhav et al., 2012; Zemla et al., 2016; Bronstein et al., 2019; Pennycook and Rand, 2019; Gronchi and Zemla, 2021) has focused on a construct based on the dual process theory of thought called cognitive reflection. It is defined as “the ability or disposition to resist reporting the response that first comes to mind” (Frederick, 2005, p. 35), and it is measured with the Cognitive Reflection Test (CRT; Frederick, 2005). The standard version of CRT is composed of three questions. Each item has an intuitive but wrong answer. Thus, the right answer requires the inhibition of the natural wrong answer and the intervention of deliberative processes in order to find the right way to correctly solve the problem. For example, one question on CRT states: A bat and a ball cost $1.10 in total. The bat costs $1.00 more than the ball. How much does the ball cost? The intuitive and automatic response is 10 cents, the difference between $1.10 and $1.00. This is the first, but potentially erroneous, response that comes to mind. Indeed, with a little reflection, an individual may realize that a response of 10 cents implies a cost for the bat of $1.10 (1$ more than the ball), for a grand total of $1.20. This clear contradiction, if noted, should induce the reader to think more carefully about the problem, concluding that the right answer is 5 cents: in this case, the bat costs $1.05 with a grand total of $1.10 as stated initially. Several ways to compute the CRT score have been proposed (Böckenholt, 2012; Sinayev and Peters, 2015; Thomson and Oppenheimer, 2016; Erceg and Bubić, 2017) in order to capture different aspects of the CRT questions. The most common calculation methods involve summing the number of correct answers (yielding a score that reflects both the use of System 2 and numerical abilities, see Sinayev and Peters, 2015) or the number of intuitive answers. More recently (Sinayev and Peters, 2015; Dorigoni et al., 2022), it has also been proposed to sum non-intuitive answers (both correct and incorrect ones) in order to measure the tendency to inhibit the initial responses that come to mind.

Within the dual process theory of thought literature, the CRT has been widely used to investigate how cognitive reflection is related to several kinds of beliefs, including paranormal and religious beliefs (Pennycook et al., 2012, 2016; Shenhav et al., 2012; Bahçekapili and Yilmaz, 2017), apparently impossible mental magic effects (Gronchi et al., 2017; Gronchi and Zemla, 2021), and fake news (Bronstein et al., 2019; Pennycook and Rand, 2019).

It is important that Frederick (2005) gave a broad definition of cognitive reflection, whereby the CRT scores reflect a mix of dispositions and abilities. Since the contribution of Frederick (2005), extensive literature has investigated if the construct of cognitive reflection can be interpreted mainly in terms of styles, abilities, or both (Campitelli and Gerrans, 2014; Stagnaro et al., 2018; Otero et al., 2022).

Also, research has developed means to induce the use of a particular thinking process. For example, adding a time constraint when performing a task (Finucane et al., 2000) makes the use of intuitive thinking more likely. Other studies (Shenhav et al., 2012) have observed that asking participants to write a paragraph about a situation where they “use their intuition/first response that comes to mind” or “careful reasoning” while solving a problem can induce the use, respectively, of intuitive or analytical thinking.

## 3. False memories and cognitive reflection

In a previous study (Gronchi et al., 2016), we employed a modified version of the DRM task, specifically the recognition test of prose memory, to investigate the hypothesis that intuitive thinking could lead to the generation of false memories. Our findings revealed a relationship between the number of intuitive responses on the CRT and the number of lures encountered. However, we were only able to provide correlational evidence linking intuitive thinking and the presence of lures based solely on recognition memory. Furthermore, we did not measure potential confounding variables.

In Gronchi et al. (2016), we hypothesized two (not mutually exclusive) possible explanations for this correlation: (i) more intuitive participants may produce more false memories because they are less effective in inhibiting lure words that come to mind (in the same way the intuitive response in the CRT is not hindered); (ii) more intuitive participants are more prone to use a familiarity heuristic. That is, given a set of potential responses (written or internally generated), they rely more on feelings of familiarity to determine if something is a real memory or not. An attempt to disambiguate these two explanations may be based on the comparison between recognition and recall. In a recall task, responses must be generated internally: according to the activation-monitoring hypothesis, both target words and lures are activated in the semantic networks, and in the monitoring phase, false memories may be generated if not sufficiently inhibited. Also, the role of inhibition in a DRM recall task has been observed by Colombel et al. (2016). In a recognition task, possible responses are already available under the eyes of the individuals. Although monitoring and inhibition play a role in this case, there is evidence that familiarity is involved in false memories in recognition tasks (Arndt, 2010; Hanczakowski et al., 2013) but not in recall tasks (Schwartz and Metcalfe, 1992; Benjamin, 2005; Malmberg, 2008).

For these reasons, we assume that the role of inhibition mechanisms should be stronger in a recall task and that the use of the familiarity heuristic should be more effective in a recognition task. Another way to differentiate the role of inhibition with the general use of a heuristic consists in employing different ways to compute the CRT score: the score that measures inhibitory control (based on the sum of non-intuitive answers, both correct and wrong, see Dorigoni et al., 2022) can contribute to highlighting the possible different roles in false memory production. In the following studies, we test the hypothesis that intuitive thinking leads one to produce more false memories. First, in a correlational study, we investigated the relation between false memories obtained with a DRM task (both recognition and recall) and the CRT, measuring several potential confounding variables (such as NFC, numerical abilities, and working memory capacity). Then, in an experimental study, we prompted either analytical or intuitive thinking [using the manipulation proposed by Shenhav et al. (2012)] to verify if the latter induces more false memories in a standard DRM task differentiating between recognition and recall.

## 4. Study 1

In our previous contribution (Gronchi et al., 2016), we found correlational evidence linking intuitive thinking, as measured by the CRT, with the number of lures encountered in recognition memory. This was achieved by utilizing a non-standard variant of the DRM task, specifically, a prose memory test. Prior to conducting an experimental study (see Study 2), our aim was to replicate these findings using a standard DRM task (Roediger and McDermott, 1995; Stadler et al., 1999), testing both recall and recognition memories. In addition, we included several other measures usually associated with the CRT (numeracy skills, working memory capacity). NFC was also incorporated because it is correlated with both the CRT and the production of false memories (Graham, 2007; Leding, 2011, 2013; Wootan and Leding, 2015). To the best of our knowledge, NFC is the only thinking-related variable that has been investigated in terms of individual differences in memory accuracy.

Also, we computed two CRT-based scores: CRT-deliberative (the sum of correct responses) and CRT-inhibition (the sum of non-intuitive responses, both correct and wrong) to differentiate the general use of analytical thinking from the inhibitory component of intuitive thinking. According to previous literature, we expect to observe the following associations:

1. In general, CRT correct scores should be positively associated with working memory capacity (Toplak et al., 2014), the NFC (Frederick, 2005), and numeracy skills (Weller et al., 2013; Baron et al., 2014; Gronchi and Zemla, 2021).
2. Assuming that the role of the inhibitory component (measured by CRT-inhibition) is stronger in the recall tasks and the familiarity heuristic (negatively associated with CRT-deliberative) is mainly involved in recognition tasks, we expect a correspondent correlation pattern depending on if false memories are determined by a lack of inhibition, a sense of familiarity, or both.
3. NFC scores should also be correlated with the number of lure words (Graham, 2007; Leding, 2011, 2013).

### 4.1. Participants

A total of 69 students (82% female) from the University of Florence were recruited for course credit. The sample mean age (in years) was 19.4 (sd = 1.3), range 18–25 (1 of unknown age). With this sample size and a Type I error rate equal to 0.05, it is possible to determine if a coefficient correlation of 0.33 differs from zero with a power of 0.80.

### 4.2. Materials and Procedure

The classic DRM paradigm (Roediger and McDermott, 1995) was administered to participants along with the Digit Span Task (Forward and Backward) and four scales: the original version of CRT, the Need for Cognition scale (NFC), and an abbreviated Numeracy Scale. All materials were presented to participants in Italian (and translated here for the reader).

CRT. The Standard version of the CRT was administered. The CRT scale includes questions that have a wrong but intuitive answer that must be inhibited to use analytical thinking to give the correct answer. Apart from the bat and ball problem described in the Introduction, the other two problems were: (i) If it takes 5 machines 5 min to make 5 widgets, how long would it take 100 machines to make 100 widgets? (Normative answer: 5 min; Intuitive answer: 100 min); (ii) In a lake, there is a patch of lily pads. Every day, the patch doubles in size. If it takes 48 days for the patch to cover the entire lake, how long would it take for the patch to cover half of the lake? (Normative answer: 47 days; Intuitive answer: 24 days). Following Dorigoni et al. (2022, see also, Böckenholt, 2012; Sinayev and Peters, 2015; Thomson and Oppenheimer, 2016; Erceg and Bubić, 2017), two scores were computed: (a) CRT-Deliberative, the number of correct answers (reflecting the degree of engagement in analytical thinking), and (b) CRT-Inhibition, the proportion of non-intuitive answers (both correct and wrong) over the total number of answers.

NFC. The 18-item version of the NFC was employed (Cacioppo and Petty, 1982). Each item was rated on a 5-point Likert scale. The scale quantifies the inclination to derive pleasure from demanding cognitive endeavors and actively engaging in them. This tendency varies among individuals. While some people exhibit low motivation and tend to avoid mentally taxing activities, others consistently pursue opportunities to partake in such tasks. Examples of items include: (i) I would prefer complex to simple problems; (ii) I like to have the responsibility of handling a situation that requires a lot of thinking; (iii) Thinking is not my idea of fun.

Numeracy scale. An abbreviated version of the Weller et al. (2013) Numeracy Scale was employed. Specifically, four items (questions 1, 2, 9, and 12) of the Weller et al. (2013) measure were administered to evaluate one’s competence in solving numerical problems. An example of an item was: Imagine that we roll a fair, six-sided die 1,000 times. Out of 1,000 rolls, how many times do you think the die would come up as an even number?

Digit span task. The test was used in two formats: Forward Digit Span, which is a measure of verbal short-term memory, and Backward Digit Span, which is a measure of central executive functioning (Collette et al., 1999). Stimuli were composed by couples of sequences of a fixed number of digits (starting from 3 digits). After a successful trial (when at least one sequence was correctly recalled), the number of digits in the sequence was increased by one. The participant’s span is the longest number of sequential digits in a successful trial. In the Forward version the sequence had to be recollected in the same order of presentation whereas in the Backward version participants were required to recollect the items in reverse order.

DRM task. Participants were presented with 18 lists of 15 semantically related words. Each list was associated with a lure (for example, the list associated with the lure “sweet” included words such as “sour,” “candy,” “sugar,” and so on). The presented lists were based on the version of the DRM task proposed by Stadler et al. (1999). A free recall test and a recognition test were administered. With regard to the recognition test, participants were required to select all the heard words from a 112-words list which included 54 target words, 18 lure words, and 36 unrelated words. So, six scores were computed, three for the Recall task (Recall Target, Recall Lure, and Recall Unrelated) and three for the Recognition task (Recognition Target, Recognition Lure, and Recognition Unrelated) as the proportion of words belonging to each category and task over the total number of words in that category.

#### 4.2.1 Procedure

Data collection was carried out over 4 days within a Psychology class. On day one, participants were requested to specify their age and gender, and a unique identification code was assigned to each participant in order to trace each individual anonymously over the 4 days. Then participants completed the standard version of the CRT questionnaire (Frederick, 2005) and the abbreviated Numeracy Scale (Weller et al., 2013) in a paper and pencil format without time limit.

On day two, the Digit Span Task was administered to assess the verbal working memory. Participants were auditorily presented with a couple of fixed series of digits of increasing length and were asked to repeat them in either the order presented (Forward span) or in reverse order (Backward span). A team of expert neuropsychologists was involved in order to administer the forward and backward tasks to each participant in two hours. Each neuropsychologist administered the task individually, reading aloud the sequences of digits and writing the result on a sheet.

On day three, a standard Deese–Roediger–McDermott (DRM) paradigm was administered to study false memories. A neuropsychologist read each list aloud with an interval of 1 s between words. Participants were instructed to memorize the words as accurately as possible and were informed that they would be tested. After each list, participants were required to write all the recalled words on a blank page for 2 min (free recall). After the subjects finished recalling the items from the last list, they turned their recall pages face down, and a filler task that lasted 10 min was proposed. In our filler task, participants were required to perform a series of simple multiplications with numbers between 1 to 9. Then, we administered the recognition task, in which participants were required to select all the heard words from a 112-word list wrote on a sheet of paper.

Finally, on day four, participants completed the NFC (Cacioppo and Petty, 1982) in a paper-and-pencil format without a time limit.

### 4.3. Results

Descriptive statistics for each computed score are reported in Table 1. Results are in line with previous literature: the mean values of CRT-Deliberative and CRT-Inhibition are similar to those observed in similar samples (Frederick, 2005; Gronchi et al., 2016; see also Supplementary material). With regard to the DRM task, results are in line with previous published studies (Roediger and McDermott, 1995; Nichols and Loftus, 2019): the recognition task was easier than the recall task, and in the former, similar percentages have been observed for Target and Lure words (69% vs. 64%). In the Recall task, 57% of the Target words were correctly remembered, whereas the percentage of lure words was about 25%. Correlations between all the variables (Table 1) are described in the next subsections.

**Table 1:** Descriptive statistics for each score and Pearson’s correlation between each couple of scores.

Variable	*n*	*M*	*SD*	1	2	3	4	5	6	7	8	9	10	11	12
1. CRT-Del.	69	1.12	1.15	—	0.38**	0.29*	0.36**	−0.07	0.31**	0.07	−0.16	0.07	0.17	−0.21	−0.08
2. CRT-Inhib.	69	2.10	1.09		—	0.43***	0.31**	0.15	0.06	0.06	−0.25*	−0.09	0.21	−0.31**	−0.02
3. SPAN	69	6.67	1.15			—	0.56***	−0.02	0.02	−0.20	−0.21	−0.03	0.05	0.00	−0.18
4. SPAN-REV	69	5.52	1.20				—	0.05	−0.06	−0.20	−0.18	0.06	0.12	−0.03	−0.26*
5. NFC	53	65.43	8.68					—	−0.10	0.14	0.10	0.13	0.06	0.06	0.24
6. Numeracy	69	2.15	1.45						—	−0.08	−0.34**	−0.16	0.22	−0.28	0.00
7. Recognition Target	69	0.69	0.11							—	0.21	0.31**	0.50***	−0.16	0.12
8. Recognition Lure	69	0.64	0.23								—	0.40**	−0.22	0.48***	0.19
9. Recognition Unrelated	69	0.06	0.05									—	0.03	0.23	−0.09
10. Recall Target	68	0.57	0.08										—	−0.21	0.02
11. Recall Lure	68	0.25	0.16											—	−0.23
12. Recall Unrelated	68	0.17	0.10												—

#### 4.3.1. CRT correlations

CRT-deliberative and CRT-inhibition showed two different patterns of correlation. The CRT-deliberative was significantly correlated with the CRT-inhibition (*r* = 0.38, *p* = 0.001), the Span-forward (*r* = 0.29, *p* = 0.017), the Span-backward (*r* = 0.36, *p* = 0.002), and the Numeracy Scale (*r* = 0.31, *p* = 0.010). On the contrary, the CRT-inhibition score was significantly correlated with the Span-forward (*r* = 0.43, *p* < 0.001), the Span-backward (*r* = 0.31, *p* = 0.010), and two scores of the DRM tasks: Recall Lure (−0.31, *p* = 0.010) and Recognition Lure (*r* = −0.25, *p* = 0.039).

#### 4.3.2. DRM scores correlation

The Recognition Target score was correlated with Recall Unrelated (*r* = 0.31, *p* = 0.009) and Recall Target (*r* = 0.50, *p* < 0.001), whereas the Recognition Lure score was correlated with Recognition Unrelated (*r* = 0.40, *p* = 0.001) and with Recall Lure (*r* = 0.48, *p* < 0.001). The Recall Unrelated score was negatively correlated with Span backward (*r* = −0.26, *p* = 0.034). Lastly, the numeracy scale was significantly correlated with Recognition Lure (*r* = −0.34, *p* = 0.005).

#### 4.3.3. Other span correlation

As expected, Span-forward was positively correlated with Span-backward (*r* = 0.56, *p* < 0.001).

### 4.4. Discussion

In Study 1, we observed a correlation between the inhibitory component measured by the CRT and the tendency to produce false memories, both in recall and recognition tasks. Contrary to previous literature (Graham, 2007; Leding, 2011, 2013; Wootan and Leding, 2015; Parker and Dagnall, 2018), we did not find an association between NFC and false memories. The distinct correlation patterns observed between the deliberative component of cognitive reflection (which showed associations with working memory and numerical skills) and the inhibitory component (which showed an association only with working memory) support the effectiveness of using both scores to highlight different aspects of cognitive reflection.

In summary, consistent with our expectations (Gronchi et al., 2016; Nichols and Loftus, 2019), intuitive thinkers provided more false memories. Correlational data suggest that this tendency depends on a decrement in inhibition efficiency rather than a general disposition toward intuition (such as the use of heuristics). In the case of recall, this difference in false memory production was not associated with individual differences in numeracy, working memory capacity, or need for cognition. Conversely, in the case of recognition, individuals with higher numerical skills made fewer recognition errors.

It should be acknowledged that the current study is characterized by limited statistical power. Future research could further explore the relationship between the DRM task and potential predictors (e.g., cognitive reflection, working memory, need for cognition, numeracy skills) by means of a multiple regression analysis.

## 5. Study 2

This paper aims to investigate whether intuitive thinking may induce a higher rate of false memory. Here we complement the correlational evidence observed in Study 1 and previous works (Gronchi et al., 2016; Nichols and Loftus, 2019) manipulating the use of the two systems of thought. Thus, we experimentally primed intuitive or analytical thinking using the approach of Shenhav et al. (2012) and then measured false memory production in a DRM task in both recall and recognition tasks. As detailed in Section 3, we put forth two possible explanations for the association between intuition and false memories. First, more intuitive individuals may be less likely to inhibit false memories that they internally generate. A second explanation (that may coexist with the first one) is that more intuitive individuals may produce more false memories because they rely more on feelings of familiarity to determine if something is a real memory or not. The comparison of recall and recognition DRM tasks can provide evidence supporting either or both explanations: the familiarity heuristic is expected to have a stronger influence in the recognition task (Arndt, 2010; Hanczakowski et al., 2013), but not in the recall task (Benjamin, 2005; Malmberg, 2008), while the inhibitory mechanism should operate conversely. Considering the observed correlation between the inhibitory component of cognitive reflection and false memory production in Study 1, our prediction was that intuitive thinkers would exhibit a higher rate of false memories in the recall task, where the inhibition of lures plays a more prominent role.

### 5.1. Participants and procedure

141 students (80% female) from the University of Florence participated in the experiment for course credit. The sample mean age (in years) was 23.7 (sd = 5.9). Following the methodology proposed by Shenhav et al. (2012; see also Gronchi and Zemla, 2021), intuitive or analytical thinking was randomly primed by means of a writing exercise. Participants were asked to recall and write about an episode of their lives in which they employed either analytical or intuitive thinking to successfully solve a problem. As in Gronchi and Zemla (2021), participants in the analytical condition read the following sentence (in Italian): “Please write a paragraph (approximately 8–10 sentences) describing a time when carefully reasoning through a situation led you in the right direction and resulted in a good outcome.” In the intuitive condition, the words “carefully reasoning through a situation” were changed to “your intuition/first instinct.” Following Shenhav et al. (2012), we excluded participants who did not write at least eight sentences (five participants, 2 from the analytical condition and 3 from the intuitive condition). Because of that, the final sample was composed of 136 students (81% Female) with a mean age of 23.8 (sd = 6.0). After the induction of analytical (67 participants) or intuitive (69 participants) thinking, participants were administered the DRM task of Experiment 1 following the same procedure. Out of the 136 participants of the final sample, 1 (analytical condition) did not complete the Recall task and then 4 (2 from the analytical condition and 2 from the intuitive condition) did not complete the Recognition task (see Supplementary material).

### 5.2. Data analysis

A mixed model approach was employed with thinking modality as a fixed variable and two random effects: individuals and the three types of memories (Target, Lure, and Unrelated) separately for recognition and recall tasks. Fisher’s Least Significant Difference *post hoc* tests were conducted to compare measures among the groups. The analyses were performed using the lme4 package (Bates et al., 2015) for the R statistical environment (version 4.1.1, R Core Team, 2021).

### 5.3. Results

In the recognition task similar percentages of correctly recognized words were observed both in the analytical and intuitive conditions (both about 66%). Participants obtained slightly lower percentages for Recognition Lure, again with negligible differences between the two conditions (62% for analytical and 63% for intuitive condition). Percentages of recognized unrelated words were very low and almost identical between analytical and intuitive thinking (about 6%). See Supplementary material for a comprehensive list of descriptive statistics.

There were no differences between analytical and intuitive condition (χ^2^_(1)_ = 0.09, *p* = 0.767). There was a significant difference among different types of recognized words (χ^2^_(2)_ = 574.92, *p* = < 0.001) whereby the proportion of unrelated words was significantly lower compared to recognized target words (*p* < 0.001) and lured words (*p* < 0.001). The number of target words and lured words were not statistically different (*p* = 0.376). The interaction between condition and different types of recognized words was not statistically significant (χ^2^_(1)_ = 0.02, *p* = 0.878).

**Figure 1:**
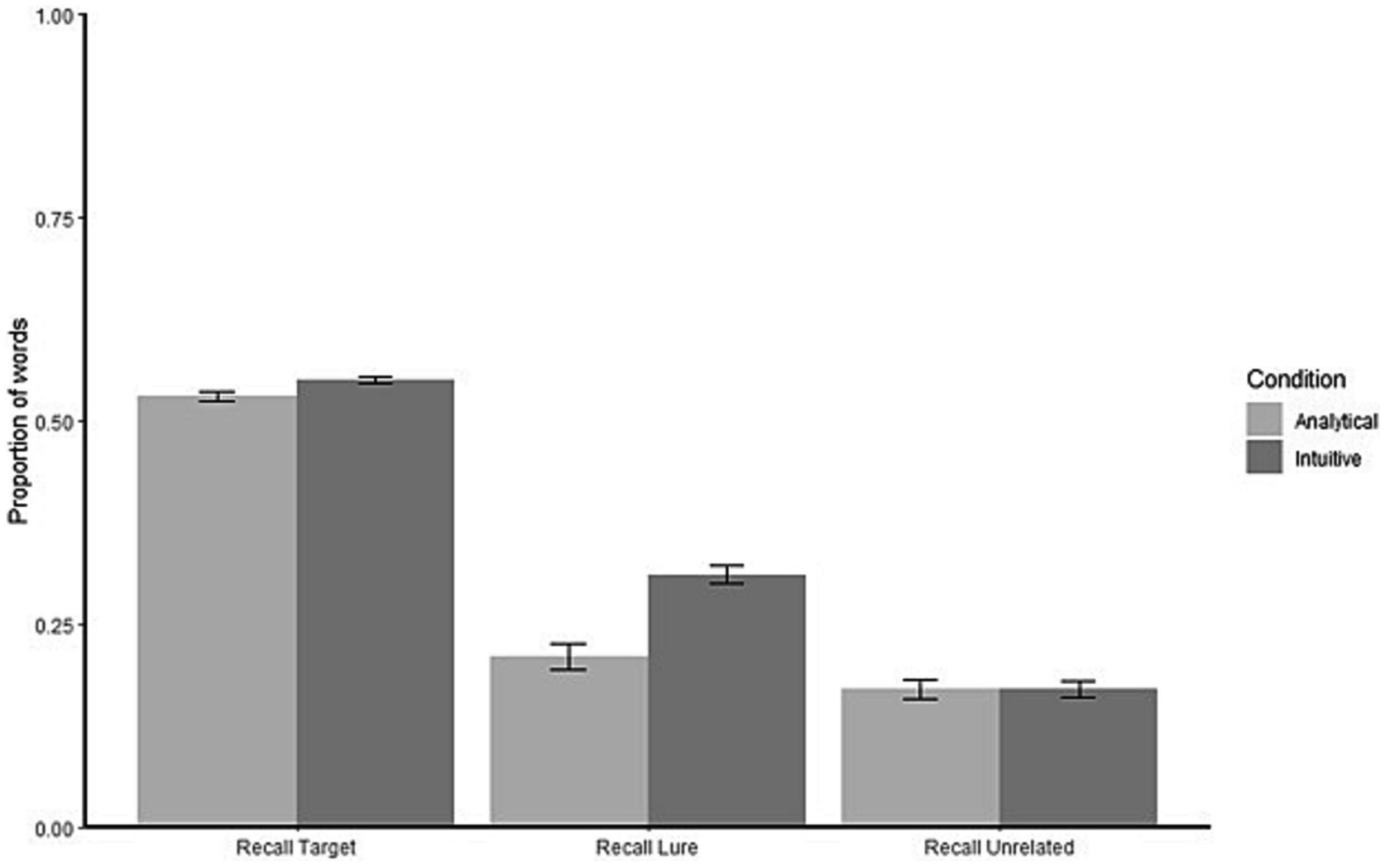
Proportion of recalled words (Target, Lure, Unrelated) for both analytical (left) and intuitive (right) condition.

With regard to the recall task (Figure 1), participants recalled 53% (Analytical condition) and 54% (Intuitive Condition) of the target words. The Recall Lure percentages were 21% for the Analytical Condition and 31% for the Intuitive condition. In both groups, the proportion of Recall Unrelated was 17%.

The main effect of Condition was statistically significant (χ^2^_(1)_ = 7.09, *p* = 0.008) as well as the main effect of different types of recalled words (χ^2^_(2)_ = 338.46, *p* < 0.001). The interaction was statistically significant (χ^2^_(1)_ = 8.28, *p* = 0.004).

Post-hoc analysis revealed that the proportion of Recall Lure was higher in the Intuitive condition compared to the Analytical condition (*p* = 0.002). No differences were found between Analytical and Intuitive conditions with regard to the Recall Target (*p* = 0.404) and Recall Unrelated score (*p* = 0.999).

In line with the DRM task expected results, Recall Target scores were higher compared to the Recall Lure and Recall Unrelated in both conditions (all *p*_s_ < 0.001). The Recall Lure score in the Intuitive condition was higher compared to both conditions’ Recall Unrelated scores (*p* < 0.001). The Recall Lure score in the Analytical condition was not statistically different compared to Recall Unrelated in the Analytical (*p* = 0.114) and Intuitive (*p* = 0.096) conditions.

### 5.4. Discussion

Results are in line with the hypothesis that intuitive thinking leads to a higher production of false memories, primarily due to a lack of inhibition mechanisms. Specifically, we observed that priming intuitive thinking increased the number of lures only in the recall task, while no effects were observed in the recognition task. Given the assumption that false memories in the recall task are mainly determined by a lack of inhibition, the results are consistent with the findings of Study 1, where only the inhibitory component of cognitive reflection was associated with false memories. Moreover, Study 2 supports the possibility that there is a causal relationship between cognitive reflection and false memories.

## 6. General discussion

The reconstructive nature of memory and the possible formation of non-veridical memories are hallmarks of memory research (Bartlett, 1932; Hemmer and Steyvers, 2009; Kiat and Belli, 2017; Baddeley et al., 2020; Frisoni et al., 2021). However, there is little research on how general thought processes may influence the formation of memories (Nichols and Loftus, 2019). In two studies, we found evidence of a relationship between cognitive reflection and false memory production, in line with previous research (Gronchi et al., 2016; Nichols and Loftus, 2019).

In a previous exploratory work (Gronchi et al., 2016), relying on the activation-monitoring explanation of false memory formation, we hypothesized that intuitive thinking could lead to more false memories due to inadequate inhibition of strongly activated lures or because of increased use of the familiarity heuristic to distinguish the memory to recollect. Exploiting the possibility to distinguish between a general tendency to use deliberation and the inhibitory component of cognitive reflection (Erceg and Bubić, 2017), in Study 1, we investigated the correlations among DRM task measures with these two components of the CRT. We found that only the inhibitory component was associated with false memory production in both recognition and recall tasks. Through manipulation of cognitive reflection obtained by priming either intuitive or analytical thinking before a DRM task, in Study 2, we confirm the centrality of the lack of inhibition in false memory production. Indeed, according to the activation-monitoring hypothesis, in the recall task, the lack of inhibition should play a central role in false memory production [as confirmed by Colombel et al. (2016)], whereas familiarity is strongly involved in the recognition task but not the recall task (Schwartz and Metcalfe, 1992; Benjamin, 2005; Malmberg, 2008; Arndt, 2010; Hanczakowski et al., 2013). On the assumption that the role inhibition component is stronger in the recall tasks whereas the familiarity heuristic is primarily involved in recognition tasks, the difference in recollected lures observed only in the recall task observed in Study 2 suggests again the centrality of a lack of inhibition in the production of false memories. As previously discussed, cognitive reflection can be thought of in terms of a mix of ability and style (Frederick, 2005). Our experiments are not able to differentiate between a lack of inhibition due to a decrease in this ability or a less marked disposition. This remains an open question for future studies.

In the recall of the DRM task of Study 1, the difference in lure production was not associated with other potential confounding variables related to numeracy, working memory capacity, or the need for cognition. Differently from expectations, we did not replicate the association between the need for cognition and false memories observed by several studies (Graham, 2007; Leding, 2011, 2013; Wootan and Leding, 2015; Parker and Dagnall, 2018). A possible explanation is that the sample of Study 1 did not guarantee enough statistical power to detect such an effect.

Although the activation monitoring hypothesis remains a credible theory, this is not the only possible explanation for false memory formation. Indeed, the Fuzzy-Trace memory theory (Reyna and Brainerd, 1995) has become increasingly central in literature on false memory (Reyna and Brainerd, 1998; Brainerd and Reyna, 2002; Reyna et al., 2016; Bialer et al., 2021). According to this theory, there are two types of memory processes. Verbatim trace represents the details of the stimulus (recollection), whereas gist trace represents the stimulus’ semantic features rather than its surface details. The false memories phenomenon could occur due to a dissociation between verbatim and gist processes in information retrieval. While true memory is sustained by both verbatim and gist processes, false memory is sustained only by gist processes that include the critical lures along with the studied items (Reyna and Brainerd, 1995; Brainerd and Reyna, 2002). Fuzzy Trace memory theory shares the same principles as the Fuzzy Trace theory of reasoning (Brainerd and Reyna, 2001), which represents an alternative account of reasoning processes compared to the dual process theory of thought (Reyna and Brainerd, 2011). A recent paper by Thompson et al. (2021) discussed the common ground and differences between the two theories. Specifically, they argue that the Fuzzy-trace theory is focused on the representations (verbatim vs. gist), while the dual-process theory of thought concentrates on the process. Keeping into account that the hypothesis that drove this work was at the process level (the relation between activation-monitoring and the dual process theory of thought distinction), our results are generally compatible with the Fuzzy Trace memory theory interpretation of false memory generation. Indeed, Reyna (2012) discussed the role of inhibition in incoherent responses when gist and verbatim representations conflict.

Overall, our work suggests that intuitive and analytical thinking can have a significant impact on false memory production, presumably because of the inhibitory component of cognitive reflection. Also, this contribution may represent a first step in deepening the relationship between higher-level thought processes and memory formation.

## Data availability statement

The raw data supporting the conclusions of this article will be made available by the authors, without undue reservation.

## Ethics statement

The study-performed according to the Declaration of Helsinki-is part of a set of behavioral and non-invasive study on face and word recognition memory processing, which were approved by the Research Committee of the University of Florence (protocol number 17245_2012). The studies were conducted in accordance with the local legislation and institutional requirements. The participants provided their written informed consent to participate in this study.

## Author contributions

GGr and SR conceived the study. GGr, SR, GGa, FG, and MV contributed to the design of the study. GGr, SR, GGa, and FG performed the experiments. SR organized the database. GGr and GGa performed the statistical analysis. GGr wrote the first draft of the manuscript. SR, FG, GGa, and MV wrote sections of it. All authors contributed to manuscript revision, read, and approved the submitted version.
