# Supplementary material for: Intuitive thinking predicts false memory formation due to a decrease in inhibitory efficiency
Source: Front Psychol. 2023 Sep 22;14:1195668. doi: 10.3389/fpsyg.2023.1195668 (PMC10556870; doi:10.3389/fpsyg.2023.1195668)
Supplement: Supplementary file 1 [file Data_Sheet_1.PDF]

<u>**Supplementary Material**</u>

	Total of correct responses	0	1	2	3
**Total of incorrect responses**	**Total of intuitive responses**				
**0**	**0**				**11**
	**1**			**9**	
	**2**		**8**		
	**3**	**8**			
**1**	**0**			**7**	
	**1**		**3**		
	**2**	**5**			
	**3**				
**2**	**0**		**1**		
	**1**				
	**2**				
	**3**				
**3**	**0**	**17**			
	**1**				
	**2**				
	**3**				

Table S.1. Study 1 CRT responses. Frequencies of total correct, intuitive and incorrect responses.

Participants	Intuitive condition	Analytical condition
141 (initial sample)	72	69
5 excluded due to invalid completion of the task	3	2
136 (final sample)	69	67
135 completed Recall tasks	69	66
131 completed Recognition tasks	67	64

Table S.2. Sample size of Study 2. Initial sample, participants excluded due to invalid completion of the thinking induction task, completed recall and recognition tasks per condition.

	Condition	Recognition Target	Recognition Lure	Recognition Unrelated	Recall Target	Recall Lure	Recall Unrelated
Sample size	Intuitive	67	67	67	69	69	69
	Analytical	64	64	64	66	66	66
Missing	Intuitive	2	2	2	0	0	0
	Analytical	3	3	3	1	1	1
Mean	Intuitive	.663	.629	.063	.545	.315	.167
	Analytical	.659	.620	.059	.535	.212	.174
SD	Intuitive	.118	.225	.044	.057	.198	.129
	Analytical	.123	.229	.042	.074	.133	.144
Minimum	Intuitive	.370	0	0	.407	.056	0
	Analytical	.259	.111	0	.311	0	0
Maximum	Intuitive	.870	1	.222	.667	1	.500
	Analytical	.926	.944	.250	.719	.500	.500

Table S.3. Study 2 descriptive statistics.
